# Optimising surgical anastomosis in ileocolic resection for Crohn’s disease with respect to recurrence and functionality: two international parallel randomized controlled trials comparing handsewn (END-to-end or Kono-S) to stapled anastomosis (HAND2END and the End2End STUDIES)

**DOI:** 10.1186/s12893-024-02340-3

**Published:** 2024-02-26

**Authors:** Anouck E. G. Haanappel, Vittoria Bellato, Christianne J. Buskens, Alessandro Armuzzi, Jarmila D. W. van der Bilt, Nanne K. H. de Boer, Silvio Danese, Eline M. L. van der Does de Willebois, Marjolijn Duijvestein, Daniëlle van der Horst, Gianluca Pellino, Milan C. Richir, Francesco Selvaggi, Antonino Spinelli, Andrea Vignali, Riccardo Rosati, Willem A. Bemelman

**Affiliations:** 1 Department of Surgery, Amsterdam UMC, University of Amsterdam, PO Box 22660, 1105 AZ Amsterdam, The Netherlands; 2 Department of Minimally invasive surgery, Tor Vergata University of Rome, Rome, Italy; 3 Department of Biomedical Sciences, Humanitas University, Pieve Emanuele – Milan, Italy; 4 Division of Colon and Rectal Surgery, IRCCS - Humanitas Research Hospital, Rozzano – Milan, Italy; 5 Department of Surgery, Flevoziekenhuis, Almere, The Netherlands; 6 Department of Gastroenterology and Hepatology, AGEM Research Institute, Amsterdam University Medical Center, Vrije Universiteit Amsterdam, Amsterdam, The Netherlands; 7 Gastroenterology and Gastrointestinal Endoscopy Unit, IRCCS Ospedale San Raffaele, Vita e Salute University, Milan, Italy; 8 Department of Gastroenterology, Radboud University Medical Center, Nijmegen, The Netherlands; 9 Crohn & Colitis NL, Woerden, The Netherlands; 10 Department of Advanced Medical and Surgical Sciences, University of Campania “Luigi Vanvitelli”, Naples, Italy; 11 Department of Surgery, University Medical Center Utrecht, Utrecht, The Netherlands; 12 Unit of Coloproctology and IBD Surgery, IRCCS Ospedale San Raffaele, Vita e Salute University, Milan, Italy; 13 Department of Gastrointestinal Surgery, IRCCS Ospedale San Raffaele, Vita e Salute University, Milan, Italy

**Keywords:** IBD, Crohn’s disease, Ileocaecal resection, Ileocolic anastomosis, CD, Handsewn anastomosis, Stapled anastomosis, Kono-S, Endoscopic recurrence, Surgical recurrence

## Abstract

**Background:**

The most common intestinal operation in Crohn’s disease (CD) is an ileocolic resection. Despite optimal surgical and medical management, recurrent disease after surgery is common. Different types of anastomoses with respect to configuration and construction can be made after resection for example, handsewn (end-to-end and Kono-S) and stapled (side-to-side). The various types of anastomoses might affect endoscopic recurrence and its assessment, the functional outcome, and costs. The objective of the present study is to compare the three types of anastomoses with respect to endoscopic recurrence at 6 months, gastrointestinal function, and health care consumption.

**Methods:**

This is a randomized controlled multicentre superiority trial, allocating patients either to side-to-side stapled anastomosis as advised in current guidelines or a handsewn anastomoses (an end-to-end or Kono-S). It is hypothesized that handsewn anastomoses do better than stapled, and end-to-end perform better than the saccular Kono-S. Two international studies with a similar setup will be conducted mainly in the Netherlands (End2End) and Italy (HAND2END). Patients diagnosed with CD, aged over 16 years in the Netherlands and 18 years in Italy requiring (re)resection of the (neo)terminal ileum are eligible. The first part of the study compares the two handsewn anastomoses with the stapled anastomosis. To detect a clinically relevant difference of 25% in endoscopic recurrence, a total of 165 patients will be needed in the Netherlands and 189 patients in Italy. Primary outcome is postoperative endoscopic recurrence (defined as Rutgeerts score ≥ i2b) at 6 months. Secondary outcomes are postoperative morbidity, gastrointestinal function, quality of life (QoL) and costs.

**Discussion:**

The research question addresses a knowledge gap within the general practice elucidating which type of anastomosis is superior in terms of endoscopic and clinical recurrence, functionality, QoL and health care consumption. The results of the proposed study might change current practice in contrast to what is advised by the guidelines.

**Trial registration:**

NCT05246917 for HAND2END and NCT05578235 for End2End (http://www.clinicaltrials.gov/).

**Supplementary Information:**

The online version contains supplementary material available at 10.1186/s12893-024-02340-3.

## Background

Crohn’s disease (CD) is a chronic inflammatory disease of the gastrointestinal tract with an unknown etiology and cure. The often transmural inflammation may be refractory to anti-inflammatory medication and can be complicated by fistula formation and strictures. Despite improvements in medical management, complicated disease leads to surgery in one-third of the patients requiring major abdominal surgery within 5 years of their diagnosis and up to 60% of patients need surgery during their disease course [1–6]. The L!RIC study demonstrated that upfront surgical management of uncomplicated ileocecal disease results in a quality of life at 1 year that is at least as good as or better than biological maintenance therapy. Surgery has been shown to be more cost-effective and associated with a lower rate of re-resection and biological use at the 5-year follow-up compared to biological maintenance therapy [7, 8].

Nonetheless, a recent prospective study by the REMIND group, reported clinical recurrence rates to be as high as 36% at 3 years and 60% at 5 years, and endoscopic recurrence (modified Rutgeerts score ≥ i2b) is reported to be as high as 70% at 1 year [9, 10].

Healthcare professionals in the field of IBD, have given considerable attention to the technical aspects of ileocolic resection with the aim of reducing recurrent Crohn’s disease after surgery. A potential role of the mesentery was highlighted, emphasizing the involvement of memory T-cells in lymphatic mesenteric tissue in perpetuating the disease at anastomosis and neo-terminal ileum [11]. Preliminary results from the SPICY trial showed that extended mesenterectomy as opposed to mesenteric sparing in ileocolic resection, does not reduce endoscopic recurrence as assessed by central reading at 6 months [12].

In a retrospective cohort study, a newly introduced anastomosis, the Kono-S, appeared to be associated with less endoscopic recurrence than the stapled side-to-side anastomosis [13]. Although the study results were notably flawed, the study reignited interest in the various types of anastomoses and their impact on disease recurrence. However, a recent prospective study demonstrated no difference in endoscopic recurrence between Kono-S and stapled side-to-side anastomosis, (47.5% vs 44.3%, respectively; *p* = 0.674), illustrating the controversies in the current literature [14].

The currently recommended anastomosis after ileocolic resection is the stapled side-to-side anastomosis due to its ease of construction and safety [15, 16]. The third type of anastomosis, the end-to-end handsewn anastomosis appears to be less popular due to its technical complexity [17]. However, a recent study comparing handsewn end-to-end with stapled side-to-side anastomosis, showed reduced healthcare consumption in favour of the end-to-end anastomosis [18]. Additionally, the authors found no difference in endoscopic recurrence rates at 2 years postoperatively (25.4% vs 39.3%, respectively; *p* = 0.112). In terms of functionality, the end-to-end anastomosis theoretically outperforms the Kono-S and stapled side-to-side anastomosis. Both have a saccular design that is presumed to be associated with luminal content stasis and consequently functional problems and recurrent Crohn’s disease [19, 20]. To date, prospective data from a randomized controlled trial comparing these three anastomoses are lacking, and it remains unclear which anastomotic technique is superior in terms of endoscopic recurrence. The aim of these two multicentre international randomized studies is to determine which anastomoses handsewn (end-to-end versus Kono-S anastomosis) or side-to-side stapled anastomosis after ileocolic resection for Crohn’s disease is superior with respect to endoscopic recurrence, gastrointestinal function, and (healthcare) costs.

## Methods/design

This study protocol is written in accordance with the SPIRIT guidelines [21, 22]. The SPIRIT checklist is provided in a supplementary file 1.

### Objectives

The primary objective of these two multicentre international randomized studies is to determine which anastomosis, handsewn (end-to-end versus Kono-S anastomosis) or side-to-side stapled anastomosis after ileocolic resection for Crohn’s disease is superior with respect to endoscopic recurrence. Secondary objectives include evaluating gastrointestinal function, postoperative morbidity, quality of life and costs.

### Study design

**Fig. 1:**
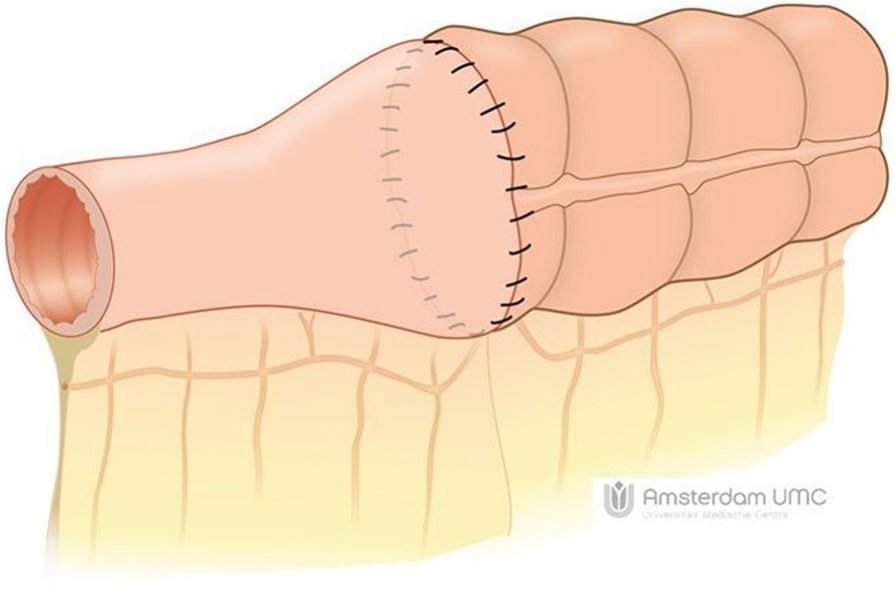
Handsewn end-to-end anastomosis

**Fig. 2:**
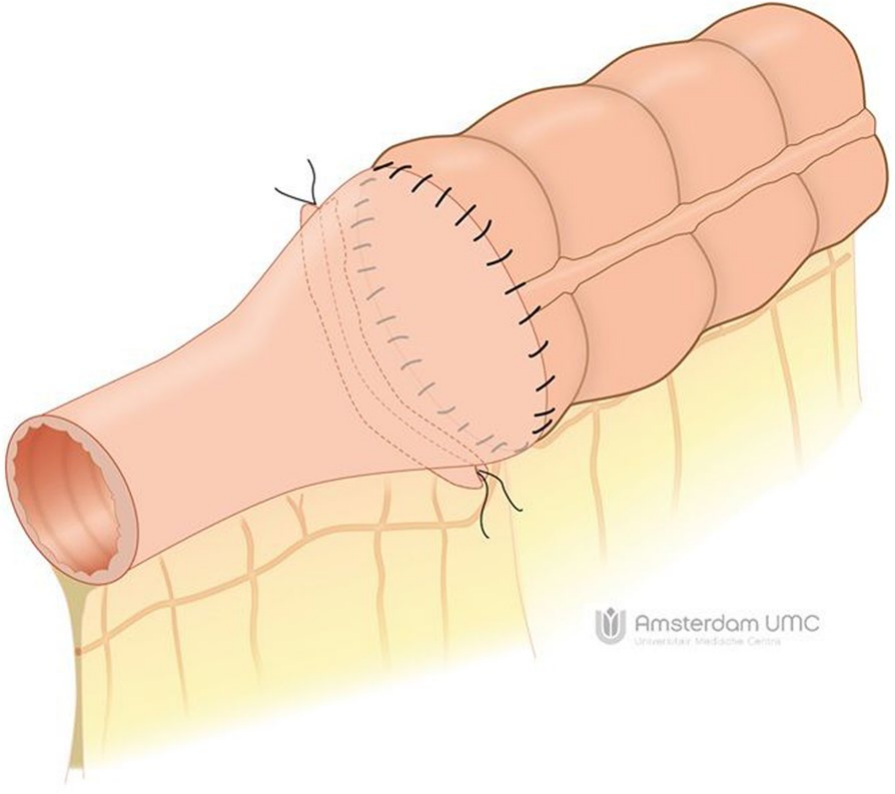
Handsewn Kono-S anastomosis

**Fig. 3:**
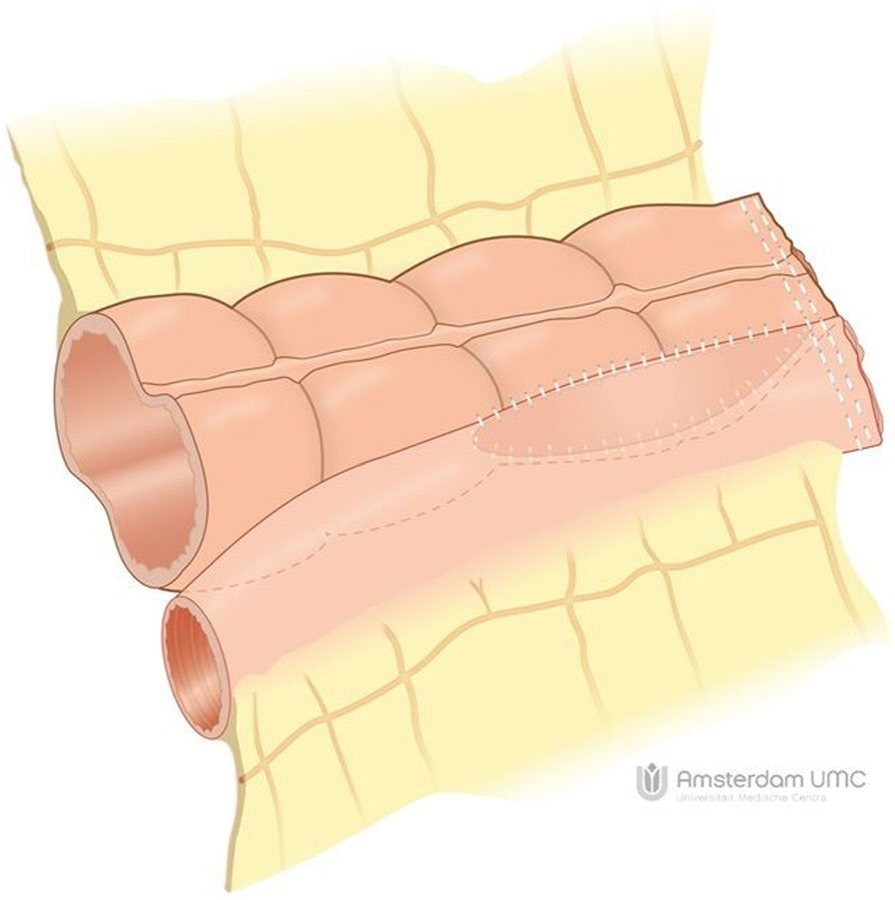
Stapled side to side anastomosis

The HAND2END and End2End studies are two separate studies with similar designs conducted respectively in both Italy and the Netherlands. Three Italian centres and 12 centres in the Netherlands will participate in this prospective study. Both studies are multicentre randomized controlled superiority trials consisting of two phases. In phase one the two handsewn anastomoses (Kono-S and end-to-end) will be compared with the side-to-side stapled anastomosis. In this phase, participants will be randomized in a 2:1 ratio between (1) handsewn end-to-end (Fig. 1) or Kono-S anastomosis (Fig. 2) and (2) stapled side-to-side anastomosis (Fig. 3). If the interim analysis shows superiority of the handsewn anastomosis, phase two of the trial will compare the end-to-end (Fig. 1) with the Kono-S anastomosis (Fig. 2). In the Netherlands, three academic and eight non-academic teaching hospitals have agreed to participate in this national initiative. This multicentre (international) collaborative will greatly facilitate the (inter)national implementation of the study results. The attached video clips will demonstrate one way of performing these anastomoses (Supplementary files 2, 3, 4).

### Study population

All patients diagnosed with CD undergoing primary ileocolic resection or resection of recurrent disease of the neoterminal ileum will be considered for inclusion. To be eligible, a subject must meet all of the following criteria: adults of either sex, age ≥ 16 years or ≥ 18 years (in the Netherlands or Italy, respectively), diagnosed with either disease of the (neo)terminal ileum or ileocolic disease (L1 and L3 disease), previously confirmed by endoscopy with an indication for resection. Patients need to have a recent update of imaging (for example, ultrasound, MR/CT enterography) before surgery, the clinical case should have been discussed at the local multidisciplinary meeting and patients need to be able to comply with the study protocol and provide written informed consent. A potential subject who meets any of the following criteria will be excluded from participation in this study: inability to provide informed consent, patient under the age of 16 or 18 years (in the Netherlands or Italy, respectively), clinically significant medical condition within 6 months before the operation (for example, myocardial infarction, active angina, congestive heart failure, or other condition that would, in the opinion of the investigators, compromise patient’s safety), history of cancer of less than 5 years that might influence the patient’s prognosis, emergent operation, pregnancy or breastfeeding.

### Ethical considerations

The trials will be conducted according to Good Clinical Practice guidelines and the principles of the Declaration of Helsinki (2013). The End2End study was approved by the Medical Ethical Committee of the Amsterdam UMC, Amsterdam (MEC 2022.0533). The protocol is registered by the Dutch Central Committee on Research Involving Human Subjects (NL81981.018.22). The HAND2END study was approved by the Medical Ethical Committee of the IRCCS San Raffaele, Milan (EC-13/06/2022, version 2.0).

### Informed consent procedure

All eligible patients referred for outpatient consultation for surgical resection will be screened for inclusion criteria during outpatient clinic visits. Patients who meet all the inclusion criteria will be informed on the details of the study and the possibility of participating in the trial. The informed consent procedure includes explanation of the study, the provision of a patient information sheet and ample time for addressing questions before signing the study consent form prior to the surgical procedure. Written informed consent is obtained by a participating surgeon, resident or trained research nurse before any study procedures. All included patients will be registered in Castor EDC and will be assigned a six-digit study number. A log of the assigned subject number will be maintained by each study site.

### Randomization

Randomization of consented study participants to either handsewn (end-to-end or Kono-S) or stapled side-to-side anastomosis will be done using an online-based system for allocation concealment (Castor EDC). Allocation concealment will be ensured, as the service will not release the randomization code until the patient has signed for informed consent and has been recruited into the trial. Randomization will be stratified for primary or repeated ileocolic resection. The randomization block sizes will not be disclosed, to ensure concealment. There is no blinding of treatment allocation for the treating surgeon. The treatment will be blinded for all other physicians (for example, gastroenterologists, central readers) and patients. A statistician blinded to treatment allocation will analyse the data.

### Study outline

#### Preoperative

Adult patients with ileocolic Crohn’s disease (CD) or recurrent disease of the terminal ileum will be screened during outpatient clinic visits. Qualified subjects expressing interest will be offered participation in the trial following multidisciplinary team discussion and will sign a written consent form if they agree to be enrolled. Medical treatment will be advised to be discontinued before surgery: glucocorticoids will be weaned to < 20 mg per day, and biologic agents and immunomodulators will be stopped at time of surgery. Prior to surgery, all patients must have had a colonoscopy with biopsy to confirm disease of the (neo)terminal ileum, and a recent update of imaging (for example ultrasound, MR or CT enterography). Baseline characteristics will be collected. Patients will all be treated in an enhanced recovery protocol.

#### Surgery

At the time of surgery, all the procedures will be initiated as a laparoscopic procedure with conversion to an open operation only if clinically indicated. Close bowel ileocolic resection is advised to preserve vascularization.

Group I: End-to-end handsewn or Kono-S anastomose. The end-to-end anastomosis is fashioned either by enlarging the small bowel diameter with an antimesenteric incision to fit the large bowel lumen or by tailored resection of a part of the staple line of the cross stapled colon. The Kono-S anastomosis is made according to the description by Kono [23]. The length of the actual anastomosis should be approximately 7 cm (Figs. 1, 2, video).

Group II: The side-to-side anastomosis is done according to local practice using a linear stapler either anisoperistaltic or isoperistaltic.

The rest of the operation will be identical. Care should be taken to ensure that, as a routine, the colon is anastomosed just beyond the ileocolic angle. Operative, postoperative and pathology data will be collected.

#### Quality control of surgical procedure

To ensure that the surgical technique is performed correctly in all centres, quality control will be conducted. A video vignette of the surgical procedure will be shared with all participating centres as an example (video). Furthermore, the participating centres will be asked to provide photos of the anastomoses and the resected specimen. Finally, each centre will provide videos of the procedure when requested.

#### Postoperative

##### Postoperative morbidity

Data on postoperative morbidity will be collected. All adverse events (AEs) and severe adverse events (SAEs) will be documented throughout the hospital stay and outpatient visits.

##### Assessment of primary endpoint (endoscopic recurrence)

All patients will undergo endoscopy with biopsies taken from the distal ileum and proximal colon on either side of the anastomosis to assess endoscopic and histologic recurrence at 6 months after surgery. Endoscopic results will be classified according to the SES-CD subdivided according to location and the (modified) Rutgeerts scoring system [24]. All colonoscopies must be video recorded according to a standard operational procedure (video). The quality of endoscopic scoring and over scoring will be assessed comparing the scoring of the local gastroenterologist with that of the central reading panel.

After endoscopic assessment at 6 months, medication can be initiated according to the severity of endoscopic recurrence according to the guidelines and the treating physician’s preference.

##### Assessment of clinical recurrence, quality of life and health care consumption

The Crohn’s Disease Activity Index (CDAI) is determined at baseline and at 6 and 12 months postoperatively based on seven-days scoring by the patient before to these visits. Quality of life questionnaires are administered at each visit (preoperative, postoperative at 3 months, at 6 months and 12 months: the EuroQol, (EQ-5D-5L), 36-Item Short Form Health Survey (SF-36), and Inflammatory Bowel Disease Questionnaire (IBDQ). All types of healthcare consumption within the first year will be recorded, including readmission, emergency room visits, examinations, medical therapy etc.

##### Medical management

Patients will be treated according to the ECCO guidelines with respect to prophylactic medication after surgery.

##### Assessment of surgical recurrence

Patients will be followed up for up to 5 years after surgery to determine the reoperation rate for recurrent disease at the anastomotic site.

### Primary and secondary outcomes

The primary outcome is endoscopic recurrence at 6 months following ileocolic resection, defined as Rutgeerts score ≥ i2b assessed by local and central reading separately and blinded for the type of anastomosis. The quality of endoscopic scoring and over scoring will be assessed comparing the local scoring by the treating gastroenterologist with the central reading scoring for the different types of anastomosis.

Secondary outcomes include postoperative morbidity, the 6 months histologic and clinical recurrence rate and the need of restarting immunosuppressive medication within the first year postoperatively for endoscopic or clinical recurrence. For long term results, patient will be followed for at least 5 years to determine reoperation rate for recurrent disease at the anastomotic site. Additionally, quality of life will be measured with IBDQ, EQ-5D and SF- 36 together with health care consumption and costs.

### Sample size calculation

The primary endpoint is the post-operative endoscopic recurrence of Crohn’s disease at 6 months.

The study is powered to detect a clinically relevant difference of 25% endoscopic recurrence at 6 months between the two randomized surgeries, 50% versus 25%. Assuming a test with continuity correction with 90% power, and an two-sided alpha-level of 0.05, in 2:1 ratio a total of 63 patients in each surgical arm will be required, for a total enrolment of 189 patients including an anticipated dropout of 10% in the Italian HAND2END study. In the Dutch End2End study, assuming a test with continuity correction with 80% power, and a two-sided alpha-level of 0.05, in 2:1 ratio a total of 55 patients per arm will be required, for a total enrolment of 165 patients, allowing for a 10% dropout.

After enrolling 189 patients in Italy, requiring 2.5 years with a 0.5-year follow-up, or 165 patients in the Netherlands, requiring 2 years with a 0.5-year follow-up, an interim analysis is done to determine whether the handsewn anastomoses are superior to stapled side-to-side anastomosis. If the interim analysis shows superiority of the handsewn anastomosis, the trial will continue with the end-to-end and the Kono-S anastomosis only. To find a reduction in endoscopic recurrence from 28 to 13% within the two handsewn groups (end-to-end and Kono-S), an additional 88 patients need to be included, for a total of 277 in the HAND2END. In Italy, a total 189 or 277 patients will be required over 3 or 4 years.. In the Dutch study, another 71 patients per group need to be included, leading to a total of 307 patients. In the End2End study, a total of 165 or 307 patients are needed over 2.5 or 4 years. If no superiority within the handsewn anastomosis is expected from the interim analysis, the study will be stopped after inclusion of either 189 patients in Italy or 165 patients in the Netherlands, plus those that are already included in the period needed to reach the 0.5-year follow-up.

The Dutch sample size has been adjusted according to the ZonMW first-round review committee’s preference, explaining why the the Dutch numbers are slightly different from those in the Italian study.

### Statistical analysis

Descriptive statistics will be used to report baseline patient and surgical variables. Baseline patient and procedure characteristics and perioperative outcome parameters are categorical, continuous and dichotomous variables, and will be presented accordingly. The chi-squared or Fisher’s exact test will be used to analyse the differences between the proportion of patients with recurrence.

#### Primary endpoint

Differences in endoscopic recurrence at 6 months between the handsewn and stapled anastomosis groups will be analysed using logistic regression, which will be corrected for stratification factors and other potential confounders. Univariate associations with the risk of recurrence will be assessed using a Cox proportional hazards model, results will be reported as a hazard ratio (HR) and 95% confidence interval (CI).

Multiple variable models will be considered in the same way depending on the number of recurrences identified. The two-sided alpha-level will be set at 0.05 for statistical significance.

Differences in QoL will be analysed using mixed-model analysis of variance for repeated measures.

At the time of analysis, the most recent version of the statistical program IBM SPSS Statistics for Windows, Armonk, NY: IBM Corp. will be used.

The statistical analysis plan will be finalized before the data is locked for analysis. Decisions will be made regarding planned subgroup analyses and how to address protocol violations and any potential baseline imbalances.

### Safety reporting

This study is considered a low-risk trial, because it involves the comparison of three well-established and commonly performed techniques in IBD surgery. No data and safety monitoring board (DSMB) will be installed. All adverse and serious adverse events (SAEs) will be monitored until they have abated or until a stable situation has been reached. The reporting procedure applies to all (S)AEs occurring up to 30 days after the surgical procedure and to any SAE that occurs after the 30-day period, if it is considered to have a reasonable possibility to be related to the protocol treatment or study participation. The study coordinator will report all SAEs to the accredited institutional review board that approved the study protocol.

### Data handling and monitoring

Each enrolled patient will be assigned a six-digit study number and all communication will occur using this number. The full name and date of birth of the patient will only be documented on the informed consent form, and these details will be securely kept in the participating hospital. In each of the participating hospitals, one surgeon acts as local investigator who is primarily responsible for execution of the study in compliance with the study protocol and ensuring the accuracy and completeness of the case report form (CRF). Data will be digitally collected and stored using the electronic data management system Castor EDC version 1.6 (www.castoredc.com). Continuous data monitoring will ensure complete and real-time prospective recording of data. Independent monitoring of the study progress and study quality will be performed by the Sponsors Clinical Monitoring Center (CMC).

### Public disclosure and publication policy

The HAND2END and End2End trials are registered at www.ClinicalTrials.gov, with registration numbers rNCT05246917 and NCT05578235, respectively. The results of both studies will be submitted separately to peer-reviewed journals, regardless of study outcomes. Additionally, the results will be presented at international conferences and disseminated to relevant surgical and IBD associations. Co-authorship will be based on the International Committee of Medical Journal Editors (ICMJE) guidelines.

## Discussion

Surgery for Crohn’s disease has been performed since the discovery of Crohn’s disease by Burrill Crohn in 1932 [25]. It is remarkable that, even after 90 years of surgical advancements, we still do not know the preferred anastomosis technique after ileocolic resection concerning safety, diagnosis of Crohn’s disease recurrence, functional outcome, and costs. The proposed trials aim to answer this unmet need.

The primary endpoint of the present study is postoperative endoscopic recurrence after 6 months. This is a crucial endpoint, because the diagnosis of endoscopic recurrence signifies disease recurrence. Disease recurrence typically necessitates the resumption of medical therapy which is in general long-term immunosuppressive or biological therapy. This entails a sequence of biological treatments for as long as the clinical symptoms do not require another surgical intervention. On the contrary, clinical recurrence has been the primary outcome in many studies in the postoperative setting, but its definition remains debated. Most importantly, there does not seem to be a strong correlation between clinical recurrence and endoscopic recurrence [26]. Furthermore, functional disorders resulting from ileocolic resection might interfere with symptoms indicating clinical recurrence.

According to ECCO guidelines, it is recommended to initiate medical therapy after surgery if the patient has one risk factor for recurrence (smoker, prior intestinal surgery, penetrating disease, perianal disease, granulomas or myenteric plexitis) [27]. All other patients should undergo endoscopy within 6–12 months to assess endoscopic recurrence. Notably, assessment of endoscopic recurrence has shown a high degree of variability (10–70%) with significant implications for both patients and society (diagnosis of recurrence and starting expensive medication) [28, 29].

The Rutgeerts score has been developed to classify endoscopic recurrence with a score of ≥2 indicating its presence. The modified Rutgeerts classification was introduced to differentiate between patients with ulcerations solely at the anastomotic line (2a) and those with ulcerations extending into the neoterminal ileum (2b). Nevertheless, endoscopic scoring remains highly variable, probably due to observer bias as evident from the significant variability between local and central readings [30].

As recommended in ECCO guidelines, the currently most popular anastomosis technique is the stapled side-to-side anastomosis. Technically, in side-to-side stapled anastomosis, both inverted and everted staple lines are present. The longitudinal staple line of both the isoperistaltic and anisoperistaltic side-to-side anastomoses is an inverted staple line, while the cross-staple line is an everted staple line. The different adaptation of the cut ends of the bowel determines the type of wound healing. Direct mucosa-to-mucosa adaptation in an everted anastomosis will result in primary wound healing, whereas the serosa-to-serosa adaptation of the longitudinal staple line will heal with secondary intent [31]. This secondary intent healing involves a process where the mucosa needs to re-epithelialize over the staple line. It was already demonstrated over 30 years ago that inverted stapled side-to-side anastomosis heal with ulcerations due to secondary wound healing where reepithelialisation is necessary and due to ischemia caused by the staples [32]. Hypothetically, these Crohn’s disease-unrelated ulcerations on the staple line could lead to systematic overscoring of endoscopic recurrence, resulting in unjustified restarting of often expensive and potential harmful drugs, reduced QOL and increased costs. This might also explain the significant variation in diagnosing endoscopic recurrence, particularly in stapled anastomoses.

Handsewn end-to-end and the Kono-S anastomoses heal with primary intent due to mucosa-mucosa adaptation on both sides and have a lower risk of overscoring endoscopic recurrence based on different types of wound healing.

To avoid anastomotic ulcerations from being misinterpreted as disease recurrence, the primary endpoint is assessed by both the local gastroenterologist and a central reading panel. A standard operating procedure (SOP) for recording the endoscopic evaluation of the anastomosis has been developed (supplementary file 5).

**Fig. 4:**
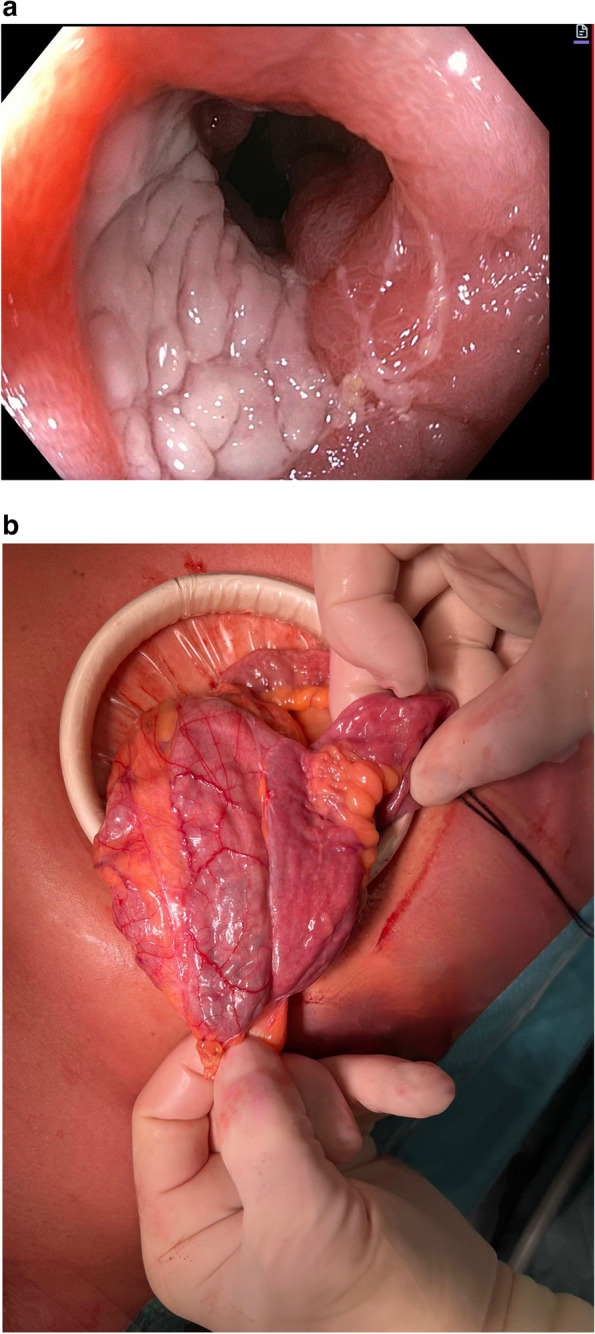
**a** Endoscopic recurrence at the inlet of the side to side anastomosis. **b** macroscopic recurrence at the inlet of the anastomosis

In addition to the potential for overscoring due to method of anastomosis construction (handsewn versus stapled), the anastomosis configuration itself might contribute to disease to recurrence. One hypothesis regarding the cause of disease recurrence is gastrointestinal luminal stasis. In this context, saccular side-to-side and Kono-S anastomoses are at risk for stasis, which may cause gastrointestinal functional disturbance and recurrent disease [19, 20]. With this regard, the disease recurrence often occurs more proximal to the inlet of the actual anastomosis rather than in the small bowel part of the anastomosis (Fig. 4a-b).

The observations of Gajendran et al. support this hypothesis [18]. In their study, they compared the side-to side stapled with the handsewn end-to-end anastomosis and noted that the former configuration resulted in worse QoL and higher healthcare consumption in the 2 years after surgery due to increased abdominal complaints and consequently diagnostic measures.

For these reasons, hypothetically the handsewn end-to-end anastomosis has the best characteristics with a lower risk of overscoring and less stasis, followed by the handsewn Kono-S (less overscoring, but potentially stasis). The expected worst anastomosis, the currently advised stapled side-to-side, potentially suffers from both overscoring and stasis.

Data from the only randomized trial focused on difference in surgical recurrence between handsewn and stapled anastomosis in ileocolic resection demonstrated the superiority of the stapled anastomosis. The recurrence rate was 9.1% in the stapled group compared to 28.8% in the handsewn group, although the number of patients in each group was only 11 vs 21 [33]. The CAST trial by McLeod, comparing stapled side-to-side with handsewn end-to-end anastomosis, found no differences in endoscopic (37.9% vs 42.5%) and clinical recurrence (22.7% vs 21.9%) in patients undergoing ileocolic resection for Crohn’s disease after a mean FU of 11.9 months [34]. A randomized trial conducted by Luglio et al. comparing the Kono-S anastomosis with the stapled side-to-side showed significant higher endoscopic recurrence rates after 6 months of the stapled anastomosis (22.2% vs 62.8%), while the clinical recurrence after 1 year did not differ (8.0% vs 18.0%) [35]. It is unclear, whether the differences in endoscopic recurrences were caused by systematic overscoring of ulcerations on the anastomotic line in the side-to-side stapled group.

The two similar studies conducted in both Italy and in the Netherlands aim to conclude which anastomosis is associated with the least recurrence of Crohn’s disease, the best function, and the least healthcare consumption. The purpose of performing two similar multicentre trials in two westernized countries, separately sponsored, is to increase the likelihood of obtaining a rapid answer, and increase the external validity and generalizability. If patient accrual is disappointing, the trails can be merged to deliver a timely result.

### Supplementary Information

**Additional file 1: Supplementary file 1.** SPIRIT checklist

**Additional file 2: Supplementary file 2.** Video Example - Handsewn end-to-end anastomosis

**Additional file 3: Supplementary file 3.** Video Example - Handsewn Kono-S anastomosis

**Additional file 4: Supplementary file 4.** Video Example - Stapled side-to-side anisoperistaltic anastomosis

**Additional file 5: Supplementary file 5.** Standard Operating Procedure (SOP) - Scoring endoscopic recurrence

## Data Availability

Data collection of both studies is still in progress. When follow up and data analysis will be finalized, data from the study will be available on reasonable request from the corresponding author.

## Abbreviations

AE: Adverse event
CD: Crohn’s disease
CDAI: Crohn’s disease activity index score
CT: Computed tomography
CRF: Case report form
ICR: Ileocolic resection
QoL: Quality of life
RCT: Randomized controlled trial
